# Development and Validation of a Radiomics Nomogram Based on ^18^F-Fluorodeoxyglucose Positron Emission Tomography/Computed Tomography and Clinicopathological Factors to Predict the Survival Outcomes of Patients With Non-Small Cell Lung Cancer

**DOI:** 10.3389/fonc.2020.01042

**Published:** 2020-07-17

**Authors:** Bin Yang, Jian Zhong, Jing Zhong, Lu Ma, Ang Li, Hengshan Ji, Changsheng Zhou, Shaofeng Duan, Qinggen Wang, Chaohui Zhu, Jiahe Tian, Longjiang Zhang, Feng Wang, Hong Zhu, Guangming Lu

**Affiliations:** ^1^Department of Medical Imaging, Affiliated Jinling Hospital, Medical School of Nanjing University, Nanjing, China; ^2^Department of Nuclear Medicine, Affiliated Jinling Hospital, Medical School of Nanjing University, Nanjing, China; ^3^GE Healthcare China, Shanghai, China; ^4^Department of Medical Imaging, Jinling Hospital, The First School of Clinical Medicine, Southern Medical University, Nanjing, China; ^5^Department of Nuclear Medicine, Peking Union Medical College Hospital, Beijing, China; ^6^Department of Nuclear Medicine, The Chinese PLA General Hospital, Beijing, China; ^7^Department of Nuclear Medicine, First People's Hospital of Nanjing, Nanjing, China

**Keywords:** non-small cell lung cancer, PET/CT, radiomics, survival outcome, risk stratification

## Abstract

**Purpose:** In this study, we developed and validated a radiomics nomogram by combining the radiomic features extracted from ^18^F-fluorodeoxyglucose positron emission tomography/computed tomography (^18^F-FDG PET/CT) images and clinicopathological factors to evaluate the overall survival (OS) of patients with non-small cell lung cancer (NSCLC).

**Patients and Methods:** A total of 315 consecutive patients with NSCLC (221 in the training cohort and 94 in the validation cohort) were enrolled in this study. A total of 840 radiomic features were extracted from the CT and PET images. Three radiomic scores (rad-scores) were calculated using the least absolute shrinkage and selection operator (LASSO) Cox regression based on subsets of CT, PET, and PET/CT radiomic features. A multivariate Cox regression analysis was performed for each rad-score combined with clinicopathological factors to determine the independent risk factors. The OS nomogram was constructed based on the PET/CT rad-score and independent clinicopathological factors. Validation and calibration were conducted to evaluate the performance of the model in the training and validation cohorts, respectively.

**Results:** A total of 144 (45.71%) women and 171 (54.29%) men with NSCLC were enrolled in this study. The PET/CT rad-score combined with the clinical model had the best C-index (0.776 and 0.789 for the training and validation cohorts, respectively). Distant metastasis, stage, carcinoembryonic antigen (CEA), and targeted therapy were independent risk factors for patients with NSCLC. The validation curve showed that the OS nomogram had a strong predictive power in patients' survival. The calibration curve showed that the predicted survival time was significantly close to the observed one.

**Conclusion:** A radiomic nomogram based on ^18^F-FDG PET/CT rad-score and clinicopathological factors had good predictive performance for the survival outcome, offering feasible, and practical guidance for individualized management of patients with NSCLC.

## Introduction

Lung cancer is a malignant tumor with the highest morbidity and mortality worldwide (1). Non-small cell lung cancer (NSCLC) is the most common pathological type of lung cancer, accounting for ~85% of all patients with lung cancer (1, 2). Considering that early signs and symptoms of NSCLC do not manifest in some patients, ~70% of patients have developed metastasis at the time of diagnosis and thus have lost the opportunity for surgical treatment (3, 4). The tumor-node-metastasis (TNM) staging system is currently the most commonly used tumor staging system worldwide and is considered to be the most valuable method for assessing the prognosis of malignant tumors (5–7). However, the TNM staging system still has several limitations when used to evaluate lung cancer prognosis in clinical practice. There are notable differences in the prognosis of tumors in the same stage, indicating that the TNM staging system cannot be used alone to fully evaluate the prognosis of patients with NSCLC. Thus, a comprehensive analysis of the TNM staging system in combination with other tumor biological characteristics that affect the prognosis of patients with NSCLC should be performed (8, 9). Therefore, determining additional effective prognostic indicators other than the TNM staging system, evaluating patients' responses to treatment at an early stage, and predicting the overall survival (OS) of patients are considered important to achieve individualized medical treatments.

With the development of genomic biology and technology, survival-related genomic characteristics have been included in the prognostic evaluation of several diseases, thereby improving the accuracy of the prognostic evaluation of several patients. However, the main limitation of these invasive technologies is that they cannot capture comprehensive information on the spatiotemporal heterogeneity of tumors (10–13). Therefore, an effective method is urgently required to comprehensively quantify the spatiotemporal heterogeneity of tumors and to evaluate the prognosis of several diseases. ^18^F-fluorodeoxyglucose positron emission tomography/computed tomography (^18^F-FDG PET/CT) is an important imaging method widely used for functional metabolic and anatomical/morphological imaging of various types of malignant tumors and metastatic lesions. ^18^F-FDG PET/CT provides not only intuitive imaging differences through image comparisons, but also several metabolic parameters to distinguish metabolically active or inactive tumor tissues. In particular, PET/CT has been widely used in clinical practice for the establishment of diagnosis, staging, efficacy monitoring, and prognostic evaluation of NSCLC (14–16). Several studies have confirmed that the FDG uptake of primary tumors is an independent risk factor for patients with early NSCLC (17, 18), but its application value in the prognostic evaluation of NSCLC is still controversial (19, 20). As an emerging and promising image analysis tool, radiomics is a non-invasive quantitative research method that can be used to convert medical images into mineable data for the identification of tumor heterogeneity. The integration of genetic pathology and imaging multimodality could improve the non-invasive quantitative analysis of tumor spatiotemporal heterogeneity and microenvironment (21, 22). Studies have shown that radiomics may have good predictive prognostic performance and decision support in oncology (23, 24). In previous studies, the texture characteristics or radiomics based on ^18^F-FDG PET/CT have been used to predict the EGFR and KRAS mutation status in patients with NSCLC, to evaluate NSCLC radiation tumor response, to predict the prognosis of patients with NSCLC after stereotactic body radiotherapy, and to stratify the risk of patients with poor prognosis. The results of these studies showed that the PET/CT-based texture characteristics or radiomics had good classification or predictive prognostic performance. Radiomics based on PET/CT may provide complementary information for predicting survival in patients with lung cancer (25–29). A nomogram is based on multivariate regression analysis and includes important influencing factors related to tumor prognosis. By constructing an intuitive graph using a statistical predictive model, the nomogram provides the numerical probability of a clinical event. The nomogram has become the focus of interest in cancer research in recent years and is considered a useful tool for quantifying risk (30–32).

Therefore, this study primarily aimed to construct a predictive model of the OS nomogram based on the radiomic features of PET/CT combined with the clinicopathological factors to predict prognosis and risk stratification as well as to determine the role of radiomic features in predicting the prognosis of NSCLC. To improve the prognostic assessment of patients with NSCLC, advancements in the areas of individualized treatment and precision medicine are necessary.

## Patients and Methods

### Patients and Clinicopathological Data

**Figure 1:**
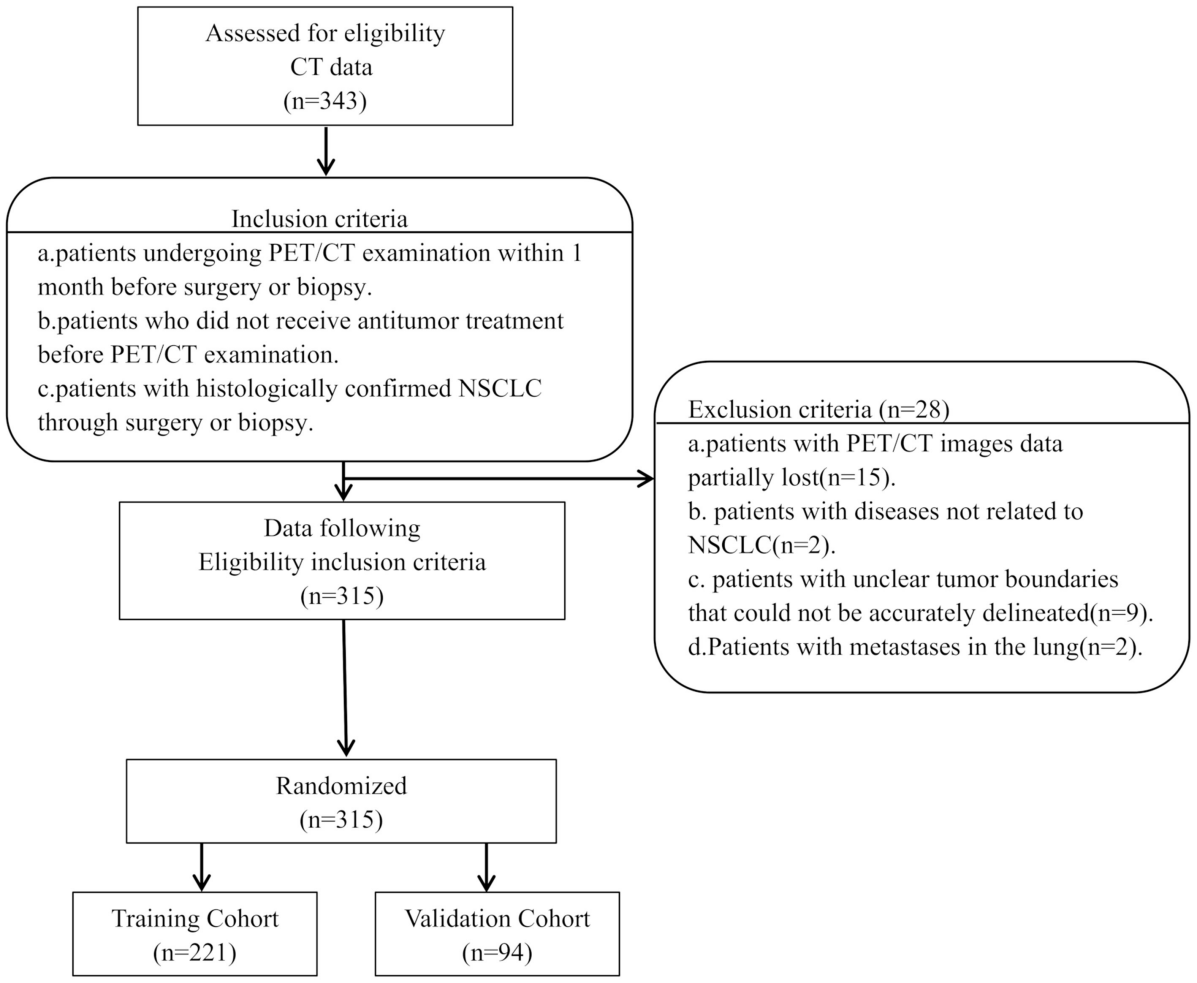
Flowchart of patient enrollment, eligibility, and exclusion criteria of the dataset.

The institutional review board of Jinling Hospital, Medical School of Nanjing University approved this retrospective study and waived the need to obtain informed consent from the patients. This was a retrospective study, and the medical records of patients between October 2007 to August 2016 were reviewed. The medical records were searched consecutively, and 343 patients who had a lung tumor as assessed by histopathological analysis were identified. Patients with the following characteristics were included in the study: (a) patients undergoing PET/CT examination within 1 month before surgery or biopsy, (b) patients who did not receive antitumor treatment before PET/CT examination, and (c) patients with histologically confirmed NSCLC through surgery or biopsy. However, patients with the following characteristics were excluded: (a) patients with partial loss of PET or CT images (*n* = 15); (b) patients with diseases not related to NSCLC (*n* = 2), (c) patients with unclear tumor boundaries that could not be accurately delineated (*n* = 9), and (d) patients with metastases in the lung (*n* = 2). The final cohort included 315 patients (Figure 1). We randomly divided the patients into the training cohort (*n* = 221) and the validation cohort (*n* = 94) with a 7:3 ratio. Clinicopathological data were obtained from the patients' medical records, which included age, sex, family history, smoking history, histological grade, lymph node metastasis, distant metastasis, and TNM stage (defined according to the eighth edition of the TNM classification and staging system by the American Joint Committee on Cancer), histologic type (adenocarcinoma, squamous cell carcinoma, or not otherwise specified [nos]), treatment methods (surgery, chemotherapy, targeted therapy, and radiotherapy), thyroid transcription factor-1 (TTF-1) level, carcinoembryonic antigen (CEA) level, tumor location, and PET/CT metabolic parameters were obtained (Table 1). The survival information of these patients was obtained through telephone calls. Follow-up data were collected from October 2007 to January 2019. The mean and median follow-up periods were 37.99 (95% confidence interval [CI], 35.464–40.522) and 36.00 (range, 20.00–52.00) months, respectively. The endpoint of this study was OS, which was defined as the period from the date of ^18^F-FDG PET/CT examination to the date of telephone follow-up or the date of the patient's death.

**Table 1:** Clinicopathological factors of patients in the training and validation cohorts.

**Characteristic**	**Training cohort *N* = 221**	**Validation cohort *N* = 94**	***P*-value**
Gender - no. (%)			1.000
Female	101 (46.0)	43 (46.0)	
Male	120 (54.0)	51 (54.0)	
Age-yr	62 (54.00-69.00)	64 (55.25-70.00)	0.672
Family history- no. (%)			0.487
No	211 (95.0)	92 (98.0)	
Yes	10 (5.0)	2 (2.0)	
T stage–no. (%)			0.879
T1	74 (33.5)	28 (29.8)	
T2	74 (33.5)	34 (36.2)	
T3	24 (10.9)	9 (9.6)	
T4	49 (22.2)	23 (24.5)	
N stage- no. (%)			0.630
N0	85 (38.5)	37 (39.4)	
N1	33 (14.9)	9 (9.6)	
N2	55 (24.9)	25 (26.6)	
N3	48 (21.7)	23 (24.5)	
M stage- no. (%)			0.439
M0	118 (53.4)	45 (47.9)	
M1	103 (46.6)	49 (52.1)	
Histologic type- no. (%)			0.445
Adenocarcinoma	210 (95.0)	92 (97.9)	
Squamous cell carcinoma	9 (4.1)	2 (2.1)	
NOS	2 (0.9)	0 (0.0)	
Surgery- no. (%)			0.054
No	133 (60.2)	68 (72.3)	
Yes	88 (39.8)	26 (27.7)	
Chemotherapy- no. (%)			1.000
No	114 (51.6)	45 (47.9)	
Yes	107 (48.4)	92 (97.9)	
Targeted therapy- no. (%)			0.584
No	168 (76.0)	68 (72.3)	
Yes	53 (24.0)	26 (27.7)	
Radiotherapy- no. (%)			0.352
No	202 (91.4)	82 (87.2)	
Yes	19 (8.6)	12 (12.8)	
Smoking status- no. (%)			0.955
No	141 (64.0)	61 (65.0)	
Yes	80 (36.0)	33 (35.0)	
Histologic grade- no. (%)			0.982
Poorly differentiated	81 (37.0)	34 (36.0)	
Moderately differentiated	102 (46.0)	43 (46.0)	
Highly differentiated	38 (17.0)	17 (18.0)	
Lymph node metastasis- no. (%)			0.663
No	85 (38.0)	33 (35.0)	
Yes	136 (62.0)	61 (65.0)	
Distant metastasis- no. (%)			0.380
No	110 (50.0)	41 (44.0)	
Yes	111 (50.0)	53 (56.0)	
Stage- no. (%)			0.847
I	52 (23.5)	22 (23.4)	
II	18 (8.1)	7 (7.4)	
III	36 (16.3)	12 (12.8)	
IV	115 (52.0)	53 (56.4)	
TTF-1- no. (%)			0.239
Negative	107 (48.0)	38 (40.0)	
Positive	114 (52.0)	56 (60.0)	
CEA	4.55 (2.30-17.50)	7.55 (3.33-37.55)	0.023
SUVmax	7.32 (4.85-10.04)	6.80 (4.07-9.67)	0.358
SUVmean	4.42 (2.96-6.48)	4.07 (2.51-6.12)	0.337
TLG(g)	31.12 (15.67-83.55)	32.05 (16.64-67.81)	0.812
MTV(cm^3^)	8.17 (4.93-16.33)	9.01 (5.14-18.76)	0.733

*CEA, carcinoembryonic antigen; MTV, metabolic tumor volume; NOS, not otherwise specified; SUVmax, maximal standard uptake value; SUVmean, mean standard uptake value; TTF-1, thyroid transcription factor-1; TLG, total lesion glycolysis*.

### PET/CT Image Acquisition and Analysis

Patients underwent PET/CT imaging (Biography 16, Siemens, Erlangen, Germany) using ^18^F-FDG synthesized by the Canadian EBCO TR19 medical cyclotron and chemical synthesis system. All PET/CT acquisitions were carried out in free breathing mode, and no steps were taken to correct for motion. The radiochemical purity was >95%. All acquisitions were carried out in a free-breathing mode. The patients fasted for 6–8 h before undergoing the scan. Patients were intravenously injected with ^18^F-FDG (5.55 MBq/kg) and underwent a whole-body PET/CT scan of the skull base to the upper part of the thigh, and the data included CT and PET scans. The CT scanning parameters were as follows: power, 120 kV; current, 140 mAs; slice thickness and spacing, 5 mm; matrix, 512 × 512; and tube rotation speed, 0.8 s/r. The PET acquisition parameters were as follows: three-dimensional at 3 min/bed; iterative algorithm; iterations, 4; subset, 8; resolution, 4.1 mm lateral, 4.6 mm axial; matrix, 128 × 128; voxel size, 5.3 × 5.3 × 5.3 mm^3^. These settings were the same for all included patients. Images were reconstructed using an iterative reconstruction method resulting in CT, PET, and PET/CT fusion images that were transferred to a post-processing workstation. We used Microsoft Viewer software (version VB10, Siemens) to calculate the metabolic parameters on the PET images. PET images were first converted to SUV images in the software without other processing methods. Then, the 3-dimensional region of interest (ROI) was manually delineated by a radiologist (W.Q.G.) to calculate the maximum standard uptake value (SUVmax, with a threshold set to 40%), mean standard uptake value (SUVmean), and metabolic tumor volume (MTV). Subsequently, the total lesion glycolysis (TLG) (TLG=SUVmean × MTV) was calculated.

### Tumor Segmentation

Our study followed and adhered to the Image Biomarker Standardization Initiative (IBSI) guidelines (33), and the software (Radiomics, Frontier, Siemens) used was IBSI-compliant. A volume of interest(VOI)was drawn semiautomatically around the tumor by a chest radiologist (Y.B., 9 years of experience) in the lung diagnosis using the radiomics prototype (Radiomics, Frontier, Siemens) and confirmed by another chest radiologist (W.Q.G., 5 years of experience). Both radiologists were blinded to the patients' clinical information. Firstly, we import CT images into radiomics prototype software (Radiomics, Frontier, Siemens). In the segmentation module, a few segmentation tools were available for semiautomatic delineation of the tumor in three dimensions. The segmentation was semiautomatically produced by drawing a line across the boundary of the tumor, then, the tool automatically find the neighboring voxels in 3D space with the same gray level through an automatic algorithm, and this is a Random Walker-based lesion segmentation for solid and subsolid lung lesions (34). The first step is to obtain a superset of voxels that may be part of the lesion. This can be implemented efficiently as a 3 D region growing starting from the center of the ROI. Then the thresholds can be fixed for lesions or determined adaptively from an analysis of the density distribution in the ROI. The region growing results in complete lesion and additionally parts of the attached vasculature. A morphological opening operation is applied to remove the vessels finally (35). If the segmentation wasn't right, the operators could correct it manually in the 3D domain using the radiomics prototype. The algorithm aimed at K-way image segmentation with given seeds indicating regions of the image belonging to the K objects(the objects to be segmented). Each seed specifies a location with a user-defined label. The algorithm labels an unseeded pixel by resolving the question: Given a random walker starting at this location, what is the probability that it first reaches each of the K seed points? It will be shown that this calculation may be performed exactly without the simulation of a random walk. By performing this calculation, we assign a K-tuple vector to each pixel that specifies the probability that a random walker starting from each un-seeded pixel will first reach each of the K seed points. A final segmentation may be derived from these K-tuples by selecting for each pixel the most probable seed destination for a random walker. By biasing the random walker to avoid crossing sharp intensity gradients, a quality segmentation is obtained that respects object boundaries (including weak boundaries) (36). And then in the radiomics module to click the computer features tool to calculate the CT radiomic features, and export the CT Masks+STL at the same time. Then we import the PET image into the software. If the tumor on the PET image is not at the same slice as the CT, we manually adjust the slice of PET image. Then, the CT Masks+STL will be imported into the software to cover the tumor on the PET image. If the CT Masks+STL does not cover the tumor, two radiologists(Y.B; W.QG) manually adjusted the CT Masks+STL through edit tools and reached a consensus to ensure that the CT Masks+STL completely covered the tumor lesions on the PET image as much as possible, and then use the same method to extract PET radiomic features. So, the 3D ROI (VOI) was delineated on CT image, and could be used by the PET image when the PET image were transformed to the CT image space using the transformation matrix obtained in PET-CT fusion.

### Radiomic Feature Extraction

**Figure 2:**
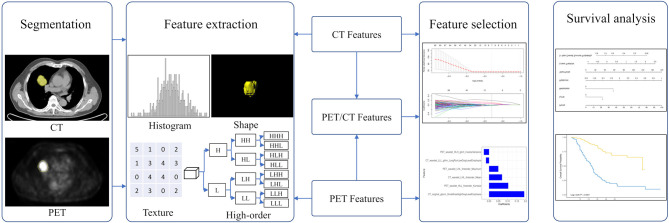
The workflow in developing a radiomic overall survival nomogram. Computed tomography (CT) and positron emission tomography (PET) images were segmented semiautomatically using the Siemens radiomics prototype. Features including histogram features, shape features, texture features, and wavelet features were extracted from CT and PET images using the software. Three rad-scores were calculated using the least absolute shrinkage and selection operator Cox regression based on subsets of CT, PET, and PET/CT radiomic features. The predictive ability of CT, PET, and PET/CT rad-scores on overall patient survival was evaluated. The overall survival nomogram was constructed based on the PET/CT rad-score and clinicopathological factors.

The Radiomic features from volumes of interest were then computed with both CT and PET images on a prototype that interfaces with the PyRadiomics library in manner similar to the 3D slicer's Radiomics plugin (34). The PyRadiomics library provides a variety of options to customize image pre-processing before feature extraction. Laplacians of Gaussian filtering, wavelet filtering, and non-linear intensity transforms were selected for image pre-processing. The feature classes contain 162 first-order features, 12 shape features, and 666 texture features. We also extracted numerous features (e.g., wavelets) that have not yet been standardized or validated by the IBSI. As a result, a total of 840 radiomic features were extracted from the CT and PET images using the software (Figure 2). The IBSI guidelines for reporting all necessary details are provided in the Supplemental Material.

### Feature Selection and Radiomics Signature Construction

Considering the redundancy of the features and to reduce model overfitting, feature engineering was performed using two methods, Spearman correlation test and the least absolute shrinkage and selection operator (LASSO) Cox regression analysis. The Spearman correlation test was initially used to reduce feature redundancy, and a cutoff value of 0.9 was adopted. A ten-fold cross-validation LASSO Cox regression method, which is suitable for the regression of high-dimensional data in survival analysis, was conducted to select the most useful predictive features from the training cohort. The specified step of the LASSO Cox analysis included determining the optimized hyperparameter λ, which ensured that the model had the least deviance. Features with non-zero coefficients were preserved. The rad-score was calculated via a linear combination of selected features weighted by their respective coefficients. Three rad-scores including the CT, PET, and PET/CT rad-scores for each patient were calculated using PET, CT, and PET/CT features, respectively (Figure 2).

### Clinicopathological Factor Analysis

Clinicopathological factors including PET/CT metabolic parameters were analyzed using a univariate Cox proportional hazards regression analysis. Factors with *p* < 0.05 were analyzed using the Kaplan–Meier curve and log-rank test. These significant factors were combined into a multivariate Cox proportional hazards regression analysis to identify independent risk factors.

### Construction and Validation of OS Nomogram

Before constructing the OS nomogram, the performance of each rad-score was evaluated using the concordance index (C-index). The largest rad-score integrated with the independent factors was used to construct the nomogram. The prognostic ability of the nomogram was evaluated in the training cohort and validated in the validation cohort. The discrimination performance of the nomogram was assessed using Harrell's C-index. The C-index ranges between 0.5 and 1.0, with 0.5 indicating a random distribution of data and 1.0 indicating the outcome of the model perfectly predicting the observed survival information. The calibration curves of the nomogram were subsequently drawn for the patients' 5-year OS. The calibration curves were used to determine the independent risk factors and also illustrated both survival probabilities predicted by the nomogram and the observed probabilities.

### Statistical Analysis

R software (version 3.5.0, www.Rproject.org) was used for statistical analysis in this study. LASSO was conducted using the “glmnet” package, while the “hdnom” package was used for the survival analysis. All statistical tests were two-sided, with a significance level of 0.05. Finally, a decision curve analysis was conducted using the “rmda” package to determine the clinical usefulness of radiomics nomogram by quantifying the net benefits at different threshold probabilities (37).

## Results

### Clinical Characteristics of Patients

The study patients were divided into two groups: the training cohort with 221 patients (120 men and 101 women) and a validation cohort with 94 patients (51 men and 43 women). There were no significant differences in sex, family history, smoking status, histological grade, lymph node metastasis, distant metastasis, TNM stage, and TTF-1 level (*p* = 0.054–1.000) between the training and validation cohorts. CEA levels were significantly different between the training and validation cohorts (*p* = 0.023). Other clinicopathological characteristics are shown in Table 1.

### Establishment of Multivariate Cox Proportional Hazards Model

Before constructing the final model, we used a multivariate Cox regression analysis to test the hazard ratio (HR) of each parameter and to determine its significance in the probability of death. The results were as follows: distant metastasis (HR, 1.94 [95% CI, 1.17–3.21]), (HR, 1.71 [95% CI, 0.81–3.61]); stage (HR, 3.24 [95% CI, 1.74–6.02]), (HR, 8.34 [95% CI, 2.28–30.56]); CEA (HR, 1.12 [95% CI, 1.03–1.21]), (HR, 1.18 [95% CI, 1.01–1.37]) and targeted therapy (HR, 0.35 [95% CI, 0.22–0.56]), (HR, 0.41 [95% CI, 0.19–0.89]) were the independent risk factors in the training and validation cohorts, respectively (Table 2).

**Table 2:** HR analysis for the different independent clinicopathological factors for clinical model.

	**Training cohort**				**Validation cohort**			
	**HR**	***p* value**	**95% CI for HR**		**HR**	***p* value**	**95% CI for HR**	
			**Lower**	**Upper**			**Lower**	**Upper**
Distant metastasis	1.94	0.010	1.17	3.21	1.71	0.162	0.81	3.61
Stage	3.24	<0.001	1.74	6.02	8.34	0.001	2.28	30.56
CEA	1.12	0.007	1.03	1.21	1.18	0.035	1.01	1.37
Targeted therapy	0.35	<0.001	0.22	0.56	0.41	0.023	0.19	0.89

*CEA, carcinoembryonic antigen; HR, hazard ratio*.

### Important Radiomic Features Selection and Calculation of the Rad-Score: Model Construction and Comparison

**Figure 3:**
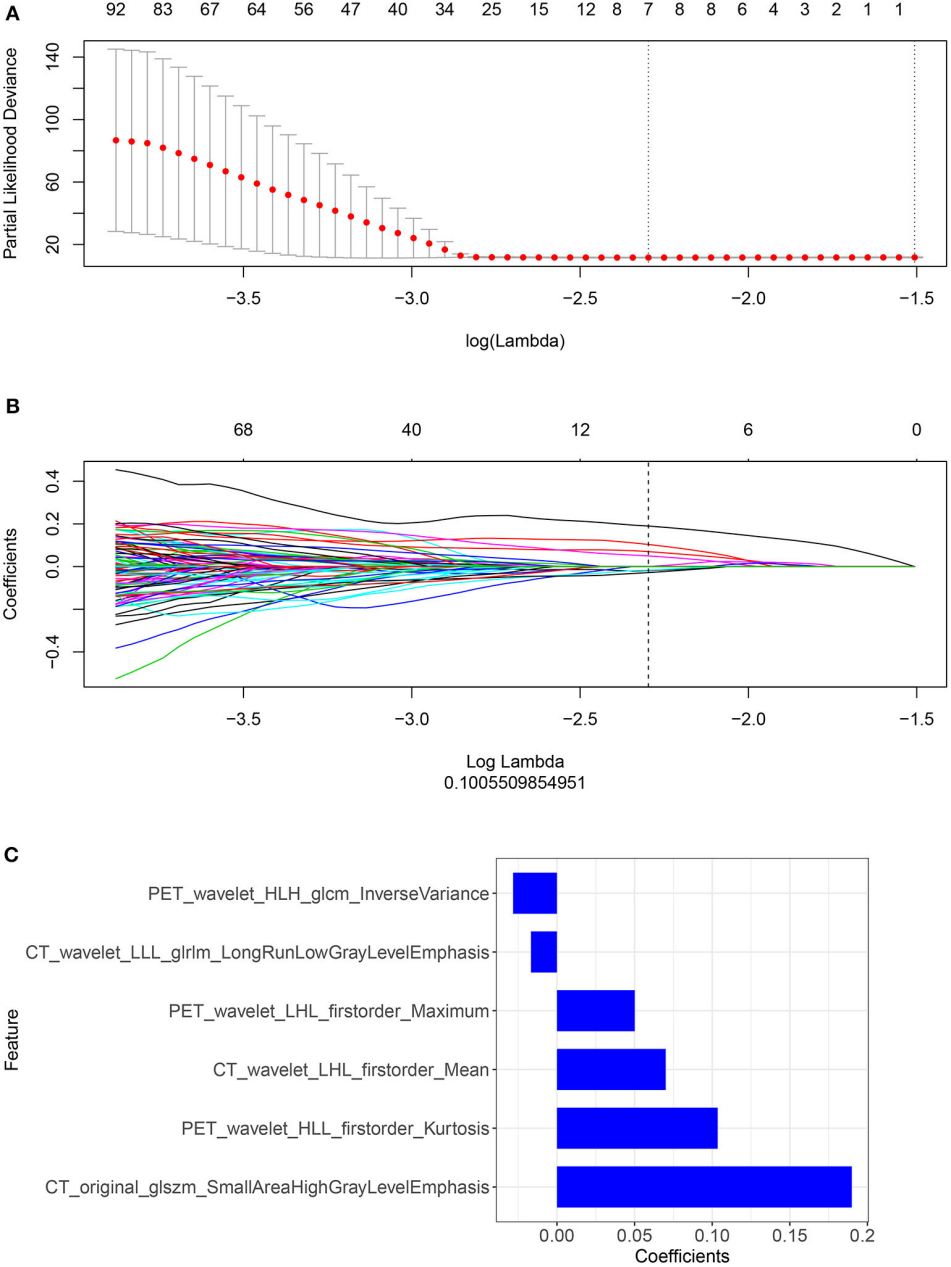
**(A,B)** Radiomic features were selected using the ten-fold cross-validation least absolute shrinkage and selection operator Cox regression model in the training cohort (number of patients: 221). The following two steps were included: determining the hyperparameter/lambda with a partial likelihood deviance as the criterion (top row) and using the optimized/lambda (the vertical dashed line) to select features with nonzero coefficients (bottom row). **(C)** A total of six important radiomic features were selected.

We performed a selection using the LASSO regression model on the PET/CT features, as shown in Figures 3A,B. To calculate the rad-score, the following six important features were selected from the 840 radiomic features, as shown in Figure 3C: PET_wavelet_HLH_glcm_Inverse Variance, CT_wavelet_LLL_glrlm_Long Run Low Gray Level Emphasis,PET_wavelet_LHL_firstorder_Maximum,CT_wavelet_LHL_firstorder_Mean, PET_wavelet_HLL_firstorder_Kurtosis, and CT_original_glszm_Small Area High Gray Level Emphasis. Subsequently, the rad-scores were calculated. The PET/CT rad-score was determined using the following formula: Rad-score=0.19 × CT_original_glszm_SmallAreaHighGrayLevelEmphasis+0.07 × CT_wavelet_LHL_firstorder_Mean+0.017 × CT_wavelet_LLL_glrlm_LongRunLowGrayLevelEmphasis+0.028 × PET_wavelet_HLH_glcm_InverseVariance+0.104 × PET_wavelet_HLL_firstorder_Kurtosis+0.05 × PET_wavelet_LHL_firstorder_Maximum−0.019.

**Figure 4:**
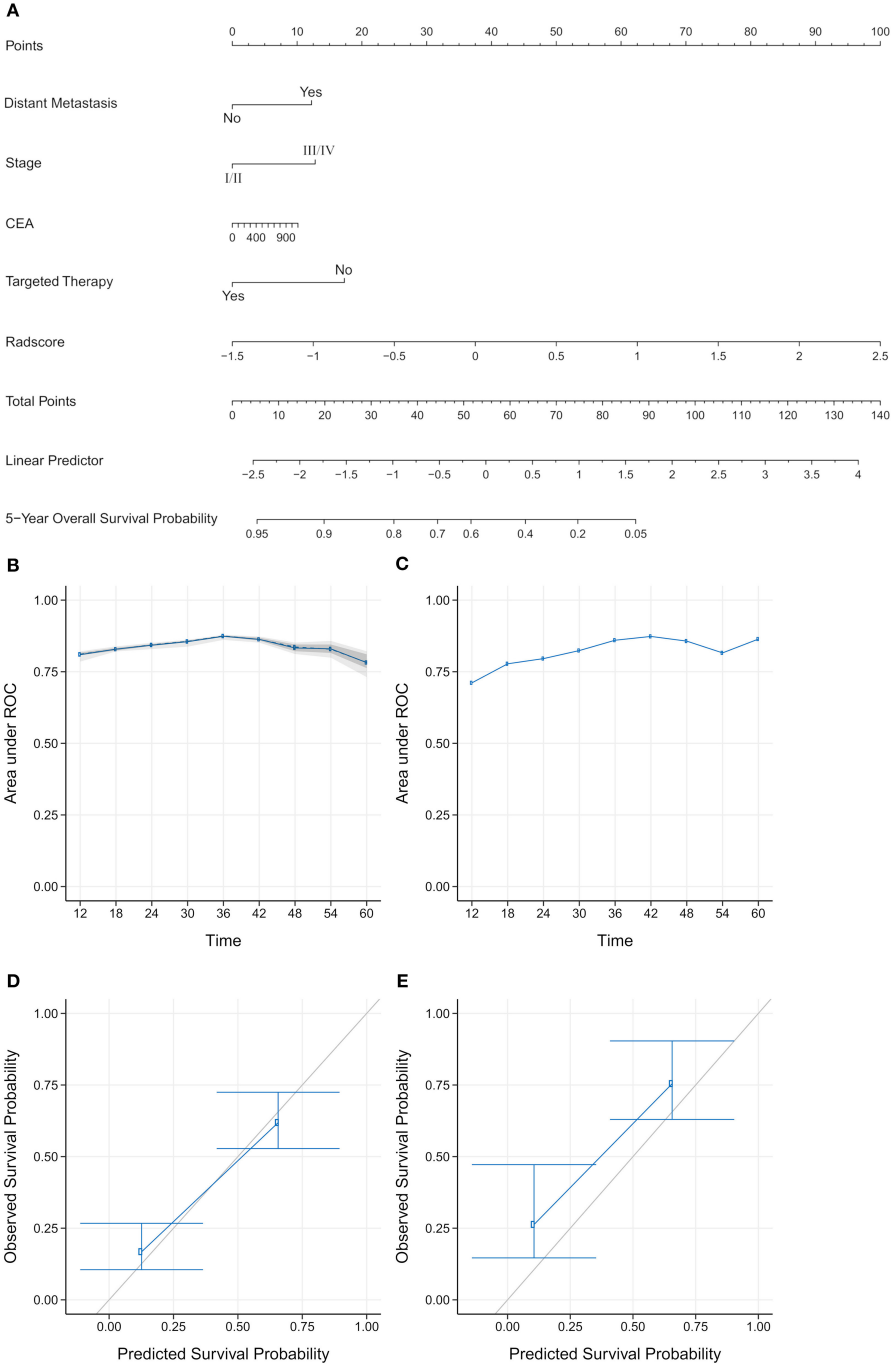
**(A)** Establishment of a comprehensive nomogram by combining the positron emission tomography/computed tomography (PET/CT) rad-score and clinicopathological factors for predicting the 5-year overall survival of patients with non-small cell lung cancer. **(B,C)** A validation analysis of the nomogram showed that the area under the curve (AUC) at five time points was obtained on the training and validation cohorts. The AUC for predicting the prognosis from 1–5 years was >0.7. With the extension of follow-up time, the predicted AUC gradually increased, indicating that the nomogram has a good performance in predicting prognosis. **(D,E)** The calibration curve was used to estimate the 5-year overall survival predicted using a nomogram. The diagonal gray line represents an ideal evaluation, and the blue line represents the performance of the nomogram. The calibration curves for the training and validation cohorts showed the calibration of two cohorts in terms of the agreement between the estimated and observed 5-year outcomes.

**Figure 5:**
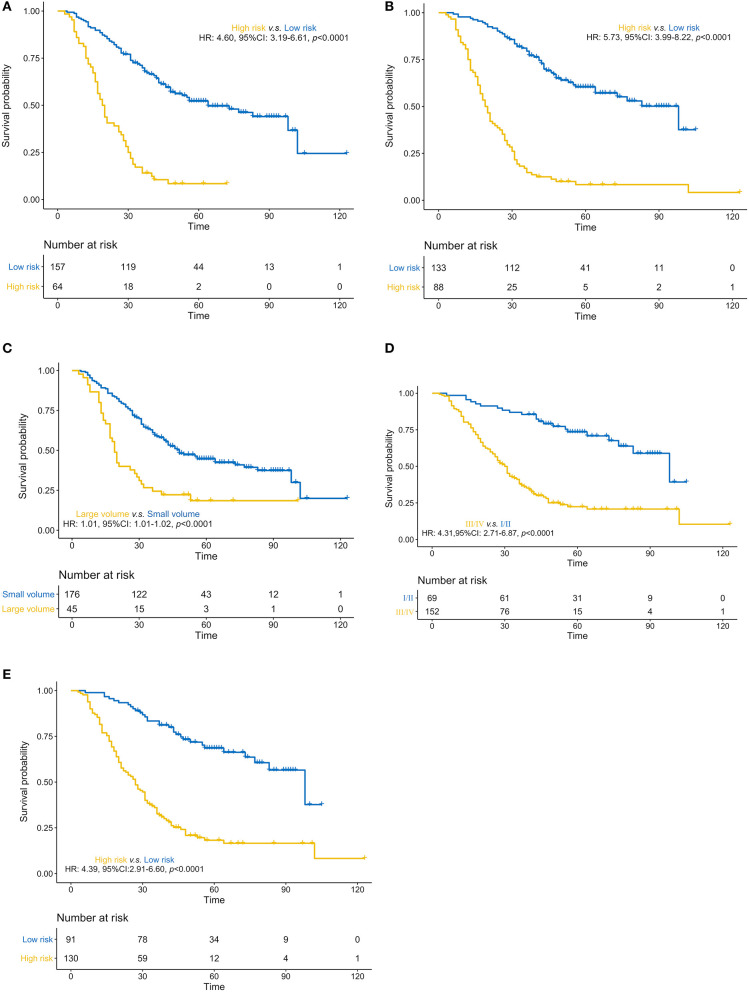
**(A–E)**. Predictive performance of the PET/CT rad-score, overall survival nomogram, tumor volume, stage, and clinical model Kaplan-Meier survival analysis of the patients in the high- and low-risk groups in the training cohort. Kaplan-Meier analysis for the PET/CT rad-score **(A)**, overall survival (OS) nomogram **(B)**, tumor volume **(C)**, stage **(D)**, and clinical model **(E)**. The patients were stratified into high- and low-risk groups based on PET/CT rad-score (**A**, *p* < 0.0001, log-rank test), OS nomogram (**B**, *p* < 0.0001, log-rank test), tumor volume (**C**, *p* < 0.0001, log-rank test), stage (**D**, *p* < 0.0001, log-rank test), and clinical model (**E**, *p* < 0.0001, log-rank test).

We constructed three rad-scores based on CT features, PET features, and PET/CT combined features. The C-index of the rad-scores is shown in Table 3. Among these three rad-scores, the CT rad-scores were 0.685 and 0.658 in the training and validation cohorts, respectively. The PET rad-score had a lower C-index (0.662 and 0.611 for the training and validation cohorts, respectively) than the CT rad-score. The PET/CT rad-score had the best C-index (0.706 and 0.661 for the training and validation cohorts, respectively). The C-index values of the clinical model with clinicopathological factors were 0.730 and 0.774 in the training and validation cohorts, respectively (Table 3). The C-index values of the TNM stage and tumor volume (0.618 and 0.635 for the training and validation cohorts, respectively) were significantly higher than that of the TNM stage (0.552 and 0.531, respectively) or tumor volume (0.607 and 0.644, respectively) alone (Table 3). A rad-score was combined with the clinicopathological factors to construct a nomogram based on LASSO, as shown in Figure 4A. The C-index (0.776 and 0.789 for the training and validation cohorts, respectively) of the PET/CT rad-score combined with the clinical model was higher than that of the clinical model without the rad-score (Table 3). The validation of the nomogram showed that it had good predictive performance, as shown in Figures 4B,C. The calibration curve showed that the predicted probability was significantly close to the actual survival time of patients, as shown in Figures 4D,E. We also analyzed the association of PET/CT rad-score, OS nomogram, tumor volume, stage, and clinical model with the survival time of patients with NSCLC using a Kaplan-Meier analysis. Figures 5A–E shows the survival probability of the patients in the high-risk or low-risk cohorts. The results of the log-rank test indicate significant discrimination between the two groups.

**Table 3:** Harrell's concordance index of different modalities.

**Modality**	**Training cohort**	**Validation cohort**
	**C-index 95% CI**	**C-index 95% CI**
CT	0.685 (0.654–0.716)	0.658 (0.593–0.723)
PET	0.662 (0.609–0.715)	0.611 (0.540–0.682)
PET/CT	0.706 (0.663–0.749)	0.661 (0.540–0.682)
Clinical model	0.730 (0.691–0.769)	0.774 (0.707–0.841)
TNM stage	0.552 (0.504–0.072)	0.531 (0.435–0.144)
Tumor volume	0.607 (0.554–0.080)	0.644 (0.560–0.127)
TNM stage and tumor volume	0.618 (0.568–0.076)	0.635 (0.548–0.132)
Radiomics nomogram (PET/CT combined with Clinical model)	0.776 (0.741–0.811)	0.789 (0.724–0.854)

*PET/CT, positron emission tomography/computed tomography*.

**Figure 6:**
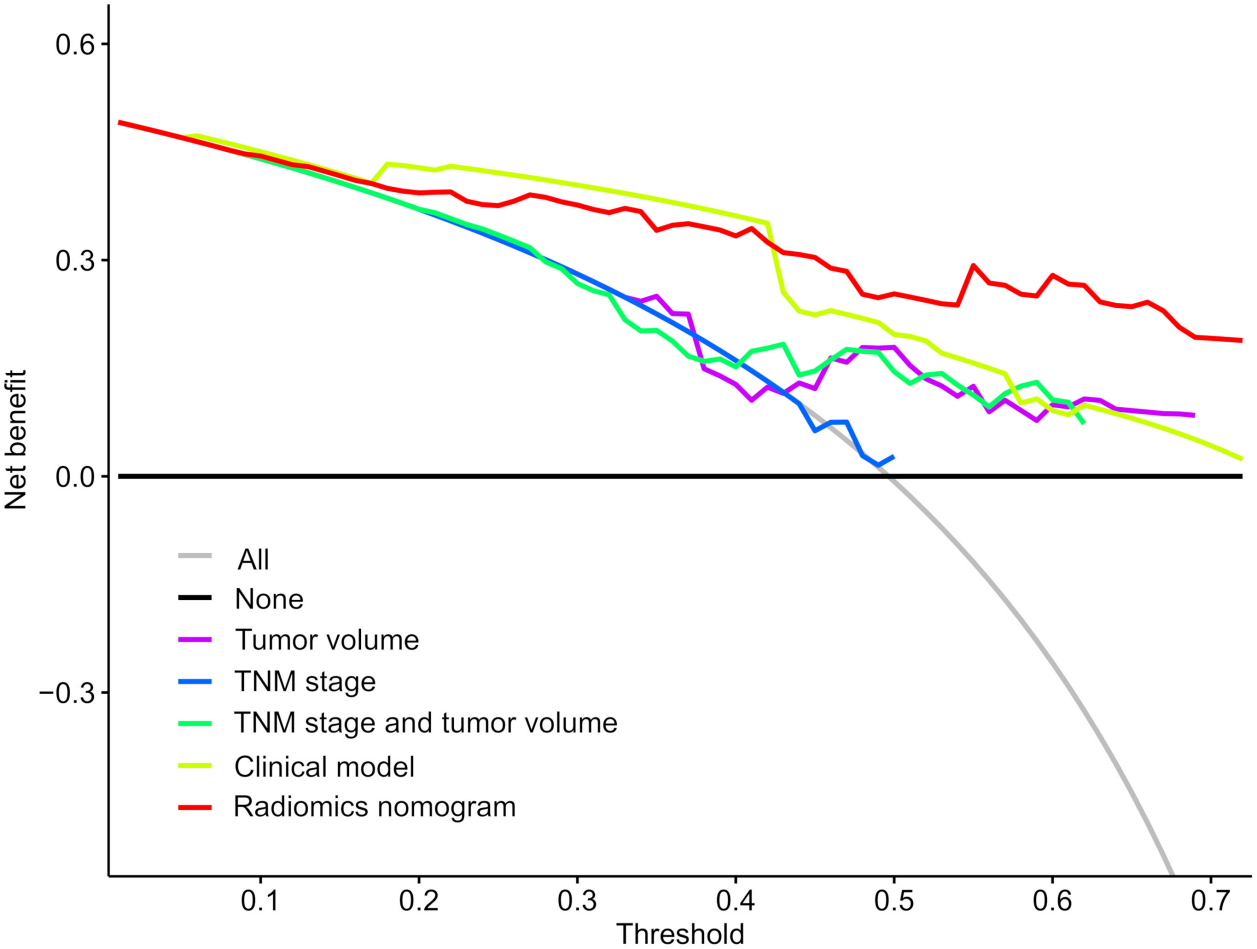
Decision curve analysis for each model. The y-axis denotes the net benefit, which was calculated using true-positive and false-positive results. The radiomics nomogram model has the highest net benefit at the threshold from 0.1 to 0.9 among all positive predictions (line labeled “All”), all negative prediction (line labeled “None”), and another 4 clinical models (line labeled “Tumor volume, tumor-node-metastasis [TNM] stage, TNM stage and tumor volume, and clinical model”).

To determine the clinical usefulness of the radiomics nomogram model, a decision curve analysis was performed. The decision curve analysis showed that the radiomics nomogram had a higher overall net benefit than 4 other clinical models (tumor volume, TNM stage, TNM stage, and tumor volume, and clinical model) across the majority of the range of reasonable threshold probabilities as shown in Figure 6.

## Discussion

In this study, we used ^18^F-FDG PET/CT radiomics to investigate the prognosis of patients with NSCLC. We extracted radiomic features from CT and PET, constructed a radiomics signature, and calculated the rad-score. Subsequently, we compared the predictive performance of CT, PET, and PET/CT rad-scores to determine the prognosis of patients with NSCLC. Considering that PET/CT has the best predictive performance among the three modalities, we further combined the PET/CT rad-score with the clinicopathological factors to predict the prognosis of patients with NSCLC. In addition, we performed Cox proportional hazards regression analysis on the clinicopathological risk factors and selected the independent risk factors related to the patient's prognosis. Finally, we constructed two prediction models based on LASSO: clinical models with and without the rad-score. In addition, 3 other clinical models were established (TNM stage, tumor volume, and tumor volume) to predict the prognosis of patients with NSCLC. Our results showed that the OS nomogram had good predictive performance for prognosis and could successfully stratify patients into high-risk and low-risk groups.

We extracted 840 radiomic features from CT, PET, and PET/CT images. To avoid redundancy and overfitting caused by the small sample size and additional radiomic features, we used the LASSO method to select important radiomic features. LASSO can be used to select biomarkers from high-dimensional radiomic features to overcome the problem of a small sample size and to select features that are most relevant to survival time (38). In addition, the LASSO method with cross-validation, as presented in this study, can be used to elegantly address issues of overfitting, collinearity, and multiple-hypothesis testing in feature selection. The LASSO method was also used to select radiomic features related to prognosis that were consistent with previous reports (39, 40). Furthermore, our prediction performance after using the LASSO method was better than the prediction performance of previous studies (38, 41, 42). Finally, we selected a total of six important radiomic features to construct CT, PET, and PET/CT radiomics signatures. The rad-scores were subsequently calculated for the three modalities to compare their predictive performances, revealing that PET/CT had the best predictive performance. Hence, we further studied the PET/CT modality, and combined the PET/CT rad-score with the clinicopathological factors that acquired good predictive performance with the C-index (0.776 and 0.789 for the training and validation cohorts, respectively). Our results showed that TNM staging was inconsistent with prognostic assessment; therefore, the prognosis cannot be predicted well. Radiomics can be used to comprehensively and quantitatively assess the spatiotemporal heterogeneity of tumors, and when combined with clinicopathological factors, the predictive performance of prognosis may be improved. According to Kirienko et al. (43), the Cox proportional hazards regression model was established based on CT, PET, and PET/CT radiomic signatures to predict the disease-free survival of patients with NSCLC. The results showed that the Cox proportional hazards regression models including radiomic features for the CT, PET, and PET/CT images had areas under the curve (AUCs) of 0.75, 0.68, and 0.68, respectively. The addition of clinicopathological risk factors to the Cox proportional hazards regression models resulted in AUCs of 0.61, 0.64, and 0.65 for the CT, PET, and PET/CT images, respectively. Mattonen et al. (44) constructed a Cox proportional hazards model that included stage and an MTV plus penumbra texture feature to predict recurrence/progression in NSCLC based on the LASSO method. The results showed that the C-index of the training and validation sets of this multivariate model were both 0.74. Wang et al. (45) used the consensus clustering method to automatically select the stable and prognostic radiomic features and subsequently constructed a multivariate Cox proportional hazards model that incorporated CT radiomic, clinical, and hematological features. These were found to be more predictive with a C-index of 0.792 and retained a C-index of 0.743 in the cross-validation analysis, therefore outperforming the radiomic, clinical, or hematological models. In addition, Dissaux et al. (27) and Oikonomou et al. (28) used PET/CT-based radiomics to predict the prognosis of patients with lung cancer who were treated with stereotactic body radiotherapy (SBRT). The results showed that radiomic features derived from PET/CT were associated with local control in patients with NSCLC undergoing SBRT, and can be used as predictors of OS, disease-specific survival, and regional control. Radiomics on PET/CT provided complementary information for the prediction of control and survival in patients with SBRT-treated lung cancer and could be helpful in clinical decision-making. The above studies showed that radiomic features were related to prognosis and had good prognostic predictive performance. Although some of the studies were multi-center studies, the sample sizes were generally small; therefore, the prediction model may have been overfitted. Our prediction model has better predictive performance and our sample size was much larger compared to previous studies. Our results indicated that the rad-score from the CT, PET, or PET/CT group has a favorable predictive power for survival. Moreover, the PET/CT rad-score had the best performance among the three rad-scores and could improve the predictive performance of the PET/CT models when combined with the clinicopathological factors. Hence, we believe that more tumor details are contained in the PET/CT entity model compared to an individual CT or PET entity model, a finding consistent with the findings of previous studies.

Additionally, we performed univariate Cox regression analysis on the clinicopathological factors to test the HR of each parameter and to determine its significance in the probability of death. The results showed that distant metastasis, stage, CEA, and targeted therapy were independent risk factors; We subsequently constructed a nomogram by combining the PET/CT rad-score and clinicopathological factors. The calibration curve showed that the predicted probability was significantly close to the actual survival time of patients. The validation of the OS nomogram showed that with the extension of follow-up time, the AUC for predicting prognosis gradually increased, and our results indicated that the OS nomogram had good predictive performance. We also evaluated the reliability of the PET/CT rad-score, OS nomogram, tumor volume, stage, and clinical model in predicting patient survival using a Kaplan–Meier analysis. The results of the Kaplan–Meier analysis demonstrated that the OS nomogram can clearly divide the patients into high-risk and low-risk groups, indicating that our nomogram had a strong predictive power in patients with high and low risks. Thus, it is considered significantly robust and reliable, and can be used as evidence for additional treatment and close follow-up in patients with poor prognosis, which is consistent with the research results of Dessroit et al. (29). In addition, the decision curve analysis demonstrated that the radiomics nomogram was superior to 4 other clinical models (tumor volume, TNM stage, TNM stage and tumor volume, and clinical model) across the majority of the range of reasonable threshold probabilities, which indicated that the radiomics nomogram added incremental value to the traditional staging system and other clinicalpathologic factors for individualized estimations. We believe that with the combination of the rad-score and clinicopathological factors to construct an OS nomogram, the predictive performance was largely improved, suggesting that the rad-score played an important role in the predictive accuracy of the OS of patients with NSCLC, a result that was consistent with the results of previous studies (46, 47). It is worth nothing that because of variations in technical parameters or inconsistent imaging parameters, a limited sample size, and heterogeneous patient characteristics, radiomic features may be insignificant in predicting prognosis in certain situations. Therefore, cohorts and validation datasets need to be evaluated, methodologies need to be standardized, and data on studies that evaluate radiomic features need to be harmonized in future studies, especially those with retrospective multi-centric datasets (48, 49).

Our study has some limitations, including the relatively small sample size and single-center cohort, the retrospective nature of the data, and the lack of external validation, which may have introduced selection bias, thereby resulting in poor model generalization and capacity. However, we plan to rapidly expand the sample size, and multi-center cohorts should be recruited for validation in the near future. Secondly, in this study, all texture matrices using 26-connectivity to find the neighboring voxels with distance 1 and 13 angles. Finally, the value of a feature is calculated for each angle separately, after which the mean of these values is used. However, it has been shown this strategy leads to less informative features compared to extracting the feature from as single matrix implementing all 13 directions, so, strategy should be implemented to merge the angle specific features into the texture matrix in future studies. Thirdly, We measured four metabolic parameters in a different way (different volume, different software) than the other radiomic features. It may lead to a bias in the comparison of the potential value of these four metrics with respect to all other features calculated by pyradiomics. And the stability and repeatability of the MTV and TLG values derived from a volume determined through a fixed threshold at 40% of maximum intensity are still controversial. So, we will try to use a consensus of several manual delineation instead of fixed thresholding to calculate tumor metabolic volume in the near future study (50, 51). In addition, all PET/CT acquisitions were carried out in free breathing mode, and no steps were taken to correct for motion may lead to extraction of features might have been suboptimal in the case of small lesions affected by motion, but also in some larger heterogeneous uptakes affected by motion blur.

In conclusion, the identified radiomic signature based on PET/CT can be potentially used as a biomarker for risk stratification of the OS in patients with NSCLC. The OS nomogram combining radiomics and clinicopathological factors for individualized OS estimation may provide more precise guidance for the accurate diagnosis and treatment of NSCLC in clinical practice.

## Data Availability Statement

The raw data supporting the conclusions of this manuscript will be made available by the authors, without undue reservation, to any qualified researcher. Requests to access the datasets should be directed to Hong Zhu, zh_zy@163.com.

## Ethics Statement

The Institutional Review Board of Jinling Hospital, Medical School of Nanjing University approved this retrospective study and waived the need to obtain informed consent from the patients.

## Author Contributions

BY conceived the idea of the study. BY, JiaZ, JinZ, LM, AL, HJ, CZhou, and QW collected the data. HZ and GL performed image analysis. BY wrote the manuscript. SD performed the statistical analysis. SD, LZ, CZhu, JT, FW, and GL edited and reviewed the manuscript. All authors contributed to the article and approved the submitted version.

## Conflict of Interest

SD was employed by GE Healthcare China. The remaining authors declare that the research was conducted in the absence of any commercial or financial relationships that could be construed as a potential conflict of interest.

## Abbreviations

C-index: Harrell's concordance index
CEA: carcinoembryonic antigen
HR: hazard ratio
MTV: metabolic tumor volume
NSCLC: non-small cell lung cancer
NOS: not otherwise specified
PET/CT: positron emission tomography/computed tomography
rad-score: radiomic score
SUVmax: maximal standard uptake value
SUVmean: mean standard uptake value
TLG: total lesion glycolysis
TNM: tumor-node-metastasis.
