# Supplementary material for: Development and Validation of a Radiomics Nomogram Based on 18F-Fluorodeoxyglucose Positron Emission Tomography/Computed Tomography and Clinicopathological Factors to Predict the Survival Outcomes of Patients With Non-Small Cell Lung Cancer
Source: Front Oncol. 2020 Jul 17;10:1042. doi: 10.3389/fonc.2020.01042 (PMC7379864; doi:10.3389/fonc.2020.01042)
Supplement: Supplementary file 1 [file Table_1.DOCX]

**The radiomics quality score:((RQS):**

Image protocol 1, Feature reduction 3, multivairable analysis 1, cutoff analysis 1, discrimination statistics 2, calibration 2, validation 4, comparison to 'gold standard', 2, potential clinical application 2, cost effective analysis 2, multi segmentation 1. Total points=21.
