# Supplementary material for: Development and Validation of a Radiomics Nomogram Based on 18F-Fluorodeoxyglucose Positron Emission Tomography/Computed Tomography and Clinicopathological Factors to Predict the Survival Outcomes of Patients With Non-Small Cell Lung Cancer
Source: Front Oncol. 2020 Jul 17;10:1042. doi: 10.3389/fonc.2020.01042 (PMC7379864; doi:10.3389/fonc.2020.01042)
Supplement: Supplementary file 2 [file Table_2.DOCX]

Reporting guidelines on the computation of radiomics features.

**Tumor segmentation and feature extraction**

We used radiomics prototype software (Radiomics, Frontier, Siemens) to segment the tumor and extract the radiomics features. All PET/CT images were transferred to this software with DICOM format and PET images were automatically converted to SUV images. Lesion segmentation tool was available in radiomics prototype software, which was a semiautomatic 3D ROI segmentation tool. The operator firstly draw a line across the boundary of the tumor, then, the tool automatically find the neighboring voxels in 3D space with the same gray level. If the segmentation wasn’t right, the operator could correct it manually. in the three-dimensional domain using the radiomic prototype. In our study, a chest radiologist(Y.B)single observer with a 9-year experience in lung diagnosis to draw the ROI on CT images using Lesion segmentation tool and confirmed by another chest radiologist(W.QG) with 5-year experience. Both radiologists were blinded to the patients’ clinical information. The 3D ROI (VOI) was delineated on CT image, and could be used by the PET image when the PET image were transformed to the CT image space using the transformation matrix obtained in PET-CT fusion. . The computation of radiomics features from VOIs were then conducted on both CT and PET images on the radiomic prototype which interfaces with PyRadiomics library in a similar manner to 3D slicer’s Radiomics plugin (34). Pyradiomics provides a tested and maintained open-source platform for easy and reproduceable radiomic feature extraction, which completely met the ISBI. The PyRadiomics library provides a variety of options to customize image pre-processing before feature extraction. The setting for extracting CT images was, imageType: original, Wavelet. Feature Class: shape, firstorder, glcm, glrlm, gldm, ngtdm, glszm. Interpolater: sitkBSpline. resamplePixelSpacing: [1, 1, 1]. padDistance: 10. binWidth: 25. Voxel Array Shift: 1000. Other settings were used default values. The PET radiomic feature used the same setting except for binWidth: 0.5, VoxelArrayShift: 0. All features formulations could be found in the following link: <https://pyradiomics.readthedocs.io/en/latest/features>. html. Totally, we had 9 image types: 1 original, 8 wavelet transformed images. 840 radiomic features (12 shape features, 18 firstorder features, 74 texture features; 12 + (18+74)*9=840 ). All texture matrices using 26-connectivity to find the neighboring voxels with distance 1 and 13 angles. Finally the value of a feature is calculated for each angle separately, after which the mean of these values is used.
